# Development and validation of a nomogram for predicting intracranial infection after intracranial aneurysm surgery

**DOI:** 10.3389/fneur.2025.1563848

**Published:** 2025-06-02

**Authors:** Yongqiang Yang, Yanli Tang, Youwen Gong

**Affiliations:** Department of Neurosurgery, Changde Hospital, Xiangya School of Medicine, Central South University (The First People’s Hospital of Changde City), Changde, China

**Keywords:** nomogram, predict, infect, intracranial, intracranial aneurysm surgery

## Abstract

**Background:**

Intracranial infection is a severe complication following intracranial aneurysm surgery, associated with higher rates of morbidity and mortality. This study aimed to develop and validate a nomogram to predict the risk for intracranial infection after intracranial aneurysm surgery. This nomogram was designed to assist clinicians in identifying high-risk patients and implementing targeted preventive measures, ultimately improving postoperative outcomes.

**Methods:**

This retrospective cohort study included patients who underwent intracranial aneurysm surgery at a single center. Data regarding potential predictors, including clinical characteristics, surgical details, and laboratory test results, were collected. Independent risk factors for intracranial infection were identified using univariate and multivariate logistic regression analyses. A nomogram was constructed on the basis of these predictors. Nomogram performance was evaluated using the area under the receiver operating characteristic curve (AUC) for discrimination, calibration plots for predictive accuracy, and decision curve analysis (DCA) for clinical utility.

**Results:**

Data from 612 patients who underwent intracranial aneurysm surgery were analyzed, with 428 and 184 patients in the training and validation cohorts, respectively. Multivariate logistic regression analysis identified pneumonia, external ventricular drainage, tracheotomy, procalcitonin, C-reactive protein, and albumin levels as independent risk factors for intracranial infections (*p* < 0.05). A nomogram, constructed on the basis of these predictors, exhibited excellent discrimination, with an AUC of 0.91 (95% confidence interval [CI] 0.88–0.93) in the training cohort and 0.89 (95% CI 0.84–0.93) in the validation cohort. DCA demonstrated that the nomogram provided a significant net clinical benefit across a range of risk thresholds, supporting its utility in clinical decision making.

**Conclusion:**

The nomogram developed was a robust and practical tool for predicting the risk for intracranial infection after intracranial aneurysm surgery. It demonstrated strong predictive accuracy and calibration, with potential applications in identifying high-risk patients and guiding individualized preventive strategies. However, validation using a broader and more diverse population is recommended to enhance the generalizability of the model.

## Introduction

Intracranial aneurysm is an aneurysm formed by abnormal expansion of arterial vessels inside the brain caused by congenital anomalies or acquired injuries (1). It is characterized by insidious onset and a high rate of disability and death (2). Surgery is often used to treat intracranial aneurysms by blocking the blood supply of the aneurysm to avoiding intracranial hemorrhage (3), and maintaining blood flow in the brain tissues (4). However, postoperative complications are also important factors affecting prognosis in this patient population (5), such as intracranial infections (ICIs) (6). Epidemiological investigations have shown that the incidence of ICIs complicating intracranial aneurysms after surgery ranges from 2.6 to 30.0%, and the mortality rate is > 30.0% (7). The occurrence of ICI (s) increases the difficulty of treatment, hospitalization costs, prolongs hospital stay, leads to readmission and/or reoperation, has serious neurological sequelae (8), and increases the risk for death (9). Therefore, early identification of high-risk factors for ICI after intracranial aneurysm surgery plays an important role in patient prognosis and reducing the risk of death (10).

Postoperatively, patients who undergo procedures for intracranial aneurysm experience more manifestations of noninfectious meningitis due to neurological impairment, fever, and cerebrospinal fluid (CSF) changes caused by underlying diseases. Furthermore, the clinical manifestations of postoperative ICIs are easily masked, making accurate diagnosis of ICI more difficult (11). Although a previous study investigated risk factors for ICI after intracranial aneurysm surgery (12), few have addressed the prediction and assessment of risk factors for ICI after aneurysm surgery using multi-index combined risk assessment. Most previous studies used logistic regression analysis and lacked effective predictive models. The nomogram presents the results of regression analysis using intuitive graphics and data to predict the probability of clinical events. Nomograms offer several advantages for predicting ICIs. By integrating multiple patient-specific and clinical variables, nomograms enable personalized risk assessment, facilitating tailored interventions for high-risk individuals (13). Their graphical representation ensures user-friendliness, enabling clinicians to easily calculate risk without requiring advanced statistical expertise. Additionally, nomograms often demonstrate superior predictive accuracy and calibration compared with conventional risk scores, enhancing their reliability in clinical practice. Accordingly, the present study aimed to develop and validate a nomogram for predicting ICI after intracranial aneurysm surgery, offering a tool to enhance risk stratification and clinical decision-making.

## Methods

### Participants

A retrospective analysis of data from 612 patients, who were admitted to First People’s Hospital of Changde City (Hunan, China) between July 2020 and January 2024, was performed. The inclusion criteria were as follows: underwent intracranial aneurysm clipping surgery; confirmed diagnosis of postoperative ICI; and complete medical records, examination data, and treatment information. Exclusion criteria were as follows: preoperative diagnosis of ICI; concurrent severe organ failure; discharge, transfer, withdrawal from treatment, or death during the operation; and incomplete medical record data.

### Data collection

In this study, basic patient information was collected within 24 h after admission, while clinical laboratory investigations were retrieved from the hospital’s electronic medical system and obtained at the time of ICI diagnosis. The collected clinical characteristics included age, length of hospital stay, sex, pneumonia, hypertension, diabetes, hyperlipidemia, coronary heart disease (CHD), external ventricular drainage (EVD), tracheotomy, and laboratory tests, including procalcitonin (PCT), Blood Lactic Acid (LaC), C-reactive protein (CRP), Bacterial Endotoxin Test (BET), albumin (Alb), blood urea nitrogen (BUN), creatinine (Cr), red blood cells (RBC), white bold cells (WBC), hemoglobin (Hb), neutrophil elastase (NE), and lymphocytes (LYM). Data entry was performed independently by 2 researchers. The dataset was validated and cleaned to ensure its accuracy and consistency. Once finalized, it was locked to prevent further modifications before statistical analysis.

### Definition of ICI

A positive CSF culture is the gold standard for diagnosing ICI. When a CSF culture is negative, an ICI can be diagnosed if the following criteria are fulfilled: fever (postoperative body temperature > 38.5°C), with other systemic infections ruled out; neurological symptoms (exclusion of other causes accompanied by symptoms such as decreased consciousness, nausea, vomiting, positive signs of meningeal irritation) and positive pathological reflexes; laboratory findings (peripheral WBC count > 10 × 10^9^/L; CSF findings include WBC count > 10 × 10^6^/L, glucose < 2.25 mmol/L, protein > 0.45 g/L, and chloride, < 120 mmol/L) (14, 15).

### Statistical analysis

Data were entered into a spreadsheet (Excel 2010, Microsoft Corp., Redmond, WA, USA), and statistical analysis was performed using SPSS version 25.0 (IBM Corp., Armonk, NY, USA) and R version 4.1 (R Core Team, R Foundation for Statistical Computing, Vienna, Austria)^1^. Categorical data are expressed as percentage (%) and analyzed using either the chi-squared test or Fisher’s exact test. For continuous data, normally distributed variables are expressed as mean ± standard deviation (SD) and compared between groups using the *t*-test, while non-normally distributed variables are expressed as median (interquartile range [IQR], i.e., P_25−_P_75_) and analyzed using the Mann–Whitney *U* test. Logistic regression was used to identify independent risk factors for ICI following intracranial aneurysm surgery, with the results expressed odds ratio (OR) and corresponding 95% confidence interval (CI). The sample was randomly split in a 7: 3 ratio, with 70% used as the modeling group and 30% as the validation group. A nomogram was generated based on the predictive model formula for visual prediction. The predictive accuracy of the model for postoperative ICI was evaluated using receiver operating characteristic (ROC) curves. The discriminative ability of the model was assessed using the area under the ROC curve (AUC) and concordance index (C-index). Model fit, calibration, and clinical utility were evaluated using the Hosmer-Lemeshow goodness-of-fit test, calibration curves, and decision curve analysis (DCA), respectively.

### Ethics approval

The study was approved by the Ethics Committee of the First People’s Hospital of Changde City (2024–025).

## Results

### Demographic data, univariate analysis, and Pearson correlation analysis

**Figure 1:**
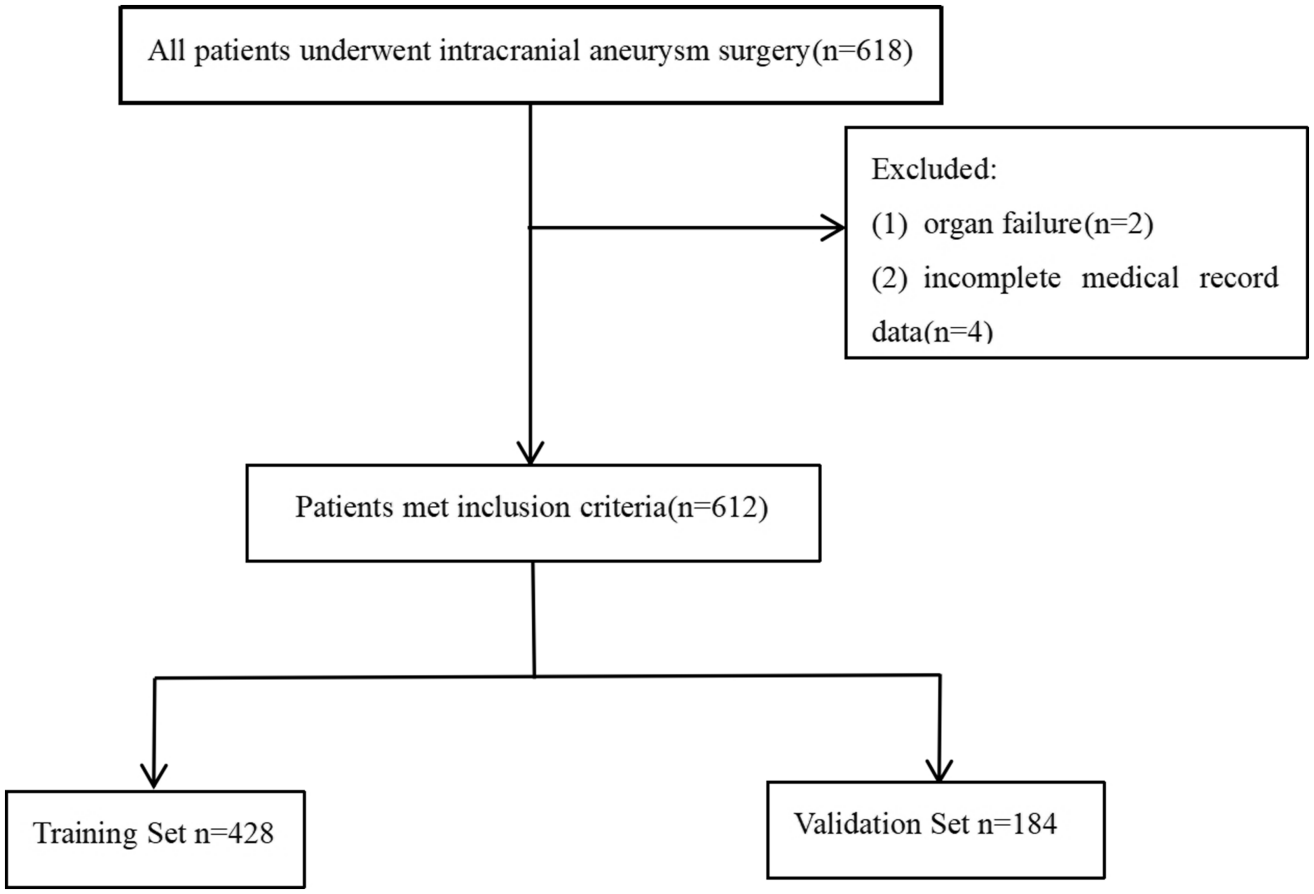
Study flow chart.

A total of 618 patients met the inclusion criteria. We excluded 2 patients with organ failure, and 4 patients with incomplete medical record data. Ultimately, a total of 612 patients were included (Figure 1). Among 612 patients who underwent surgery for intracranial aneurysms, 68 developed postoperative ICI, corresponding to an incidence rate of 11.1%. Most patients in the study population were elderly (mean [± SD] age, 60.55 ± 9.51 years) and female (55.4%). In terms of clinical characteristics, there was a statistically significant difference between the non-ICI group and the ICI group in terms of length of stay, sex, pneumonia, hypertension, hyperlipidemia, CHD, EVD, and tracheotomy (*p* < 0.05). The differences in age and diabetes status were not statistically significant (*p* > 0.05) (Table 1). According to Pearson correlation analysis, there was no significant correlation of ICI with Age, Diabetes, Cr, RBC, Hb, NE, and LYM. Regarding laboratory investigations comparing the non-ICI and ICI groups, univariate analysis revealed that PCT (*p* < 0.001), LaC (*p* < 0.001), CRP (*p* < 0.001), BET (*p* < 0.001), Alb (*p* < 0.001), BUN (*p* < 0.001), and WBC (*p* < 0.001) were associated with an increased odds of ICIs. However, there were no statistical differences between the 2 groups in terms of Cr, RBC, Hb, NE, or LYM (*p* > 0.05) (Table 1).

**Table 1:** Univariate analysis and Pearson correlation analysis results of the ICI following intracranial aneurysm surgery.

Variables	Total (*n* = 612)	No infection group (*n* = 544)	Infection group (*n* = 68)	*χ*^2^/*t*/*Z*	Pearson value	*p*
Age	60.55 ± 9.51	60.50 ± 9.67	60.99 ± 8.21	−0.51	0.016	0.607
Length of stay	19.00 (13.00, 29.00)	18.00 (12.00, 28.00)	25.50 (20.75, 40.25)	−4.68	0.135**	<0.001
Gender				21.61	0.317**	<0.001
Male	273 (44.61)	273 (50.18)	40 (58.82)			
Female	339 (55.39)	271 (49.82)	28 (41.18)			
Pneumonia				71.12	0.341**	<0.001
No	377 (61.60)	367 (67.46)	10 (14.71)			
Yes	235 (38.40)	177 (32.54)	58 (85.29)			
Hypertension				20.51	0.183**	<0.001
No	265 (43.30)	253 (46.51)	12 (17.65)			
Yes	347 (56.70)	291 (53.49)	56 (82.35)			
Diabetes				2.01	−0.057	0.156
No	523 (85.46)	461 (84.74)	62 (91.18)			
Yes	89 (14.54)	83 (15.26)	6 (8.82)			
Hyperlipidemia				11.38	0.136**	<0.001
No	504 (82.35)	458 (84.19)	46 (67.65)			
Yes	108 (17.65)	86 (15.81)	22 (32.35)			
CHD				7.81	0.113**	0.005
No	559 (91.34)	503 (92.46)	56 (82.35)			
Yes	53 (8.66)	41 (7.54)	12 (17.65)			
EVD				24.47	0.200**	<0.001
No	508 (83.01)	466 (85.66)	42 (61.76)			
Yes	104 (16.99)	78 (14.34)	26 (38.24)			
Tracheotomy				20.09	0.181**	<0.001
No	480 (78.43)	441 (81.07)	39 (57.35)			
Yes	132 (21.57)	103 (18.93)	29 (42.65)			
PCT	0.41 (0.19, 0.64)	0.37 (0.17, 0.58)	0.89 (0.62, 1.08)	−10.39	0.509**	<0.001
LaC	8.89 (5.47, 11.81)	9.69 (6.49, 12.15)	1.12 (0.75, 1.46)	−13.45	−0.650**	<0.001
CRP	33.73 (19.13, 48.38)	33.29 (18.56, 47.33)	43.27 (23.57, 57.29)	−3.32	0.153**	<0.001
BET	0.84 (0.34, 1.37)	0.90 (0.52, 1.43)	0.16 (0.14, 0.23)	−11.92	−0.436**	<0.001
Alb	37.40 (32.53, 43.79)	39.06 (34.36, 44.61)	30.27 (27.96, 31.73)	−11.01	−0.427**	<0.001
BUN	7.43 (5.26, 9.63)	7.66 (5.41, 10.16)	6.35 (3.94, 7.82)	−4.61	−0.194**	<0.001
Cr	63.00 (47.00, 79.10)	64.00 (47.00, 81.00)	59.82 (52.07, 69.24)	−1.18	−0.060	0.238
RBC	3.64 (3.02, 4.24)	3.65 (2.99, 4.34)	3.50 (3.22, 3.93)	−1.02	−0.052	0.307
WBC	10.48 (8.24, 12.87)	10.40 (8.00, 12.83)	11.34 (9.84, 13.93)	−3.51	0.176**	<0.001
Hb	94.32 (87.03, 102.72)	94.22 (86.65, 102.49)	96.00 (89.00, 103.00)	−1.15	0.048	0.250
NE	8.57 (6.22, 11.01)	8.48 (6.16, 11.00)	9.06 (6.85, 11.12)	−1.19	0.047	0.235
LYM	1.38 (0.88, 1.90)	1.39 (0.89, 1.91)	1.32 (0.87, 1.78)	−0.96	−0.041	0.335

CHD, coronary heart disease; CRP, C-reactive protein; BET, Bacterial Endotoxin Test; Alb, albumin; PCT, Procalcitonin; LaC, Blood Lactic Acid; BUN, Blood Urea Nitrogen; Cr, creatinine; RBC, red blood cell; WBC, white blood cell; Hb, Hemoglobin; NE, neutrophilic granulocyte; LYM, lymphocyte.

*χ*^2^, Chi-square test value; *t*, *t*-test value; *Z*, Mann–Whitney *U* test value.

** *p* < 0.01.

### Multivariate analysis

All factors that were statistically significant (i.e., *p* < 0.05) in the univariate analysis were included in the logistic multivariate analysis, including length of hospital stay, sex, pneumonia, hypertension, hyperlipidemia, CHD, EVD, tracheotomy, PCT, LaC, CRP, BET, Alb, BUN, and WBC. A stepwise method was used to select the variables. Results revealed that pneumonia (OR 4.904 [95% CI 3.410–6.397]), EVD (OR 5.883 [95% CI 4.513–7.253]), tracheotomy (OR 5.983 [95% CI 4.619–7.348]), PCT (OR 4.332 [95% CI 3.048–5.616]), CRP (OR 6.862 [95% CI 5.127–8.597]), and Alb (OR 4.679 [95% CI 3.610–5.747]) were independent risk factors (Table 2). A risk prediction model was established for ICI based on these 6 predictors, which were independently associated with the odds of ICI according to logistic regression analysis.

**Table 2:** Multivariate logistic regression analysis for ICI after intracranial aneurysm surgery.

Variable	*β*	SE	Wald *χ*^2^	*p*	OR (95% CI)
Pneumonia	5.942	2.323	2.561	0.010	4.904 (3.410 ~ 6.397)
EVD	4.112	2.095	1.973	0.049	5.883 (4.513 ~ 7.253)
Tracheotomy	1.789	0.669	6.607	0.002	5.983 (4.619 ~ 7.348)
PCT	7.966	3.303	2.414	0.016	4.332 (3.048 ~ 5.616)
CRP	0.096	0.047	2.242	0.025	6.862 (5.127 ~ 8.597)
Alb	−1.052	0.501	−2.090	0.037	4.679 (3.610 ~ 5.747)

*β*, regression coefficient; CI, confidence interval.

### Nomogram establishment and evaluation

**Figure 2:**
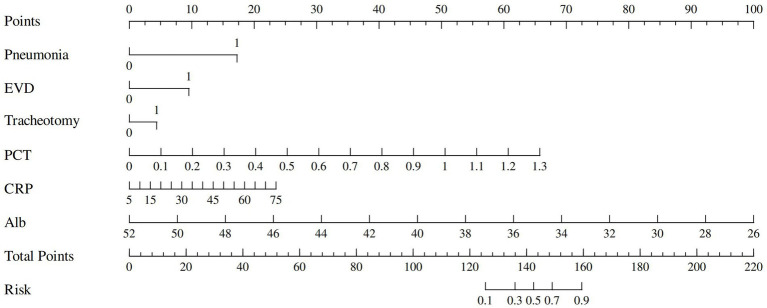
Nomogram for predicting risk of ICI.

Based on the results of multivariate logistic regression analysis, 6 independent risk factors were identified as predictors of ICI after intracranial aneurysm surgery. A nomogram was developed using these predictors to provide a visual, user-friendly tool for risk prediction (Figure 2). Each predictor was assigned a score according to its regression coefficient, with higher scores reflecting a greater contribution to ICI risk. The total score was calculated by summing the individual scores for all predictors and mapping to the corresponding probability of ICI.

**Figure 3:**
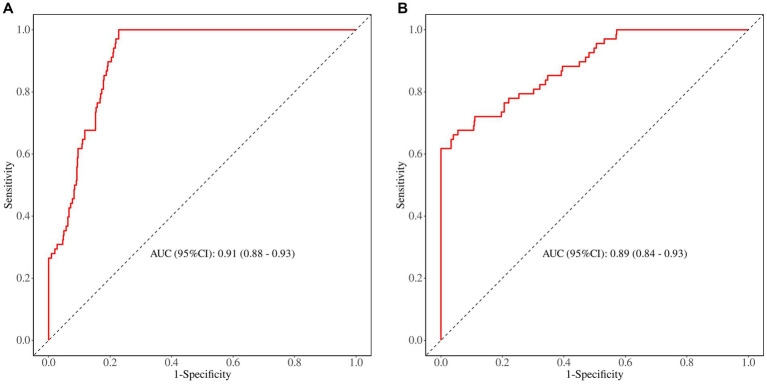
**(A)** The ROC curve in the training set. **(B)** The ROC curve in the validation set.

**Figure 4:**
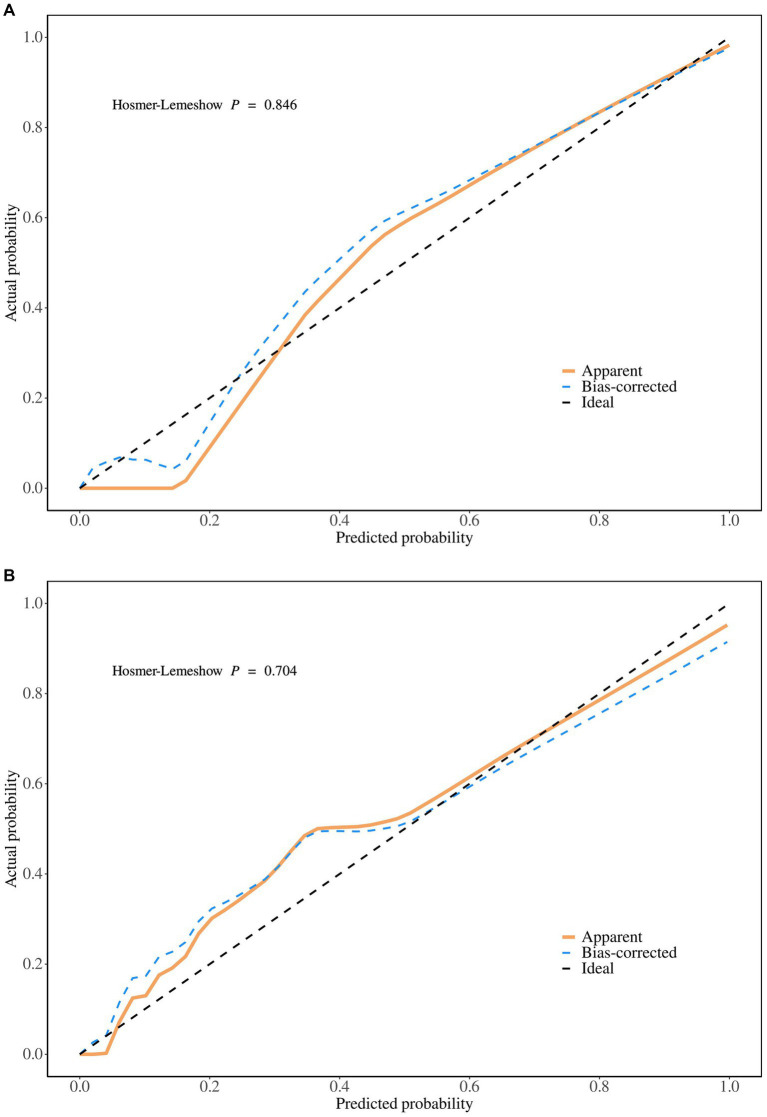
**(A)** The calibration plot in the training set. **(B)** The calibration plot in the validation set.

**Figure 5:**
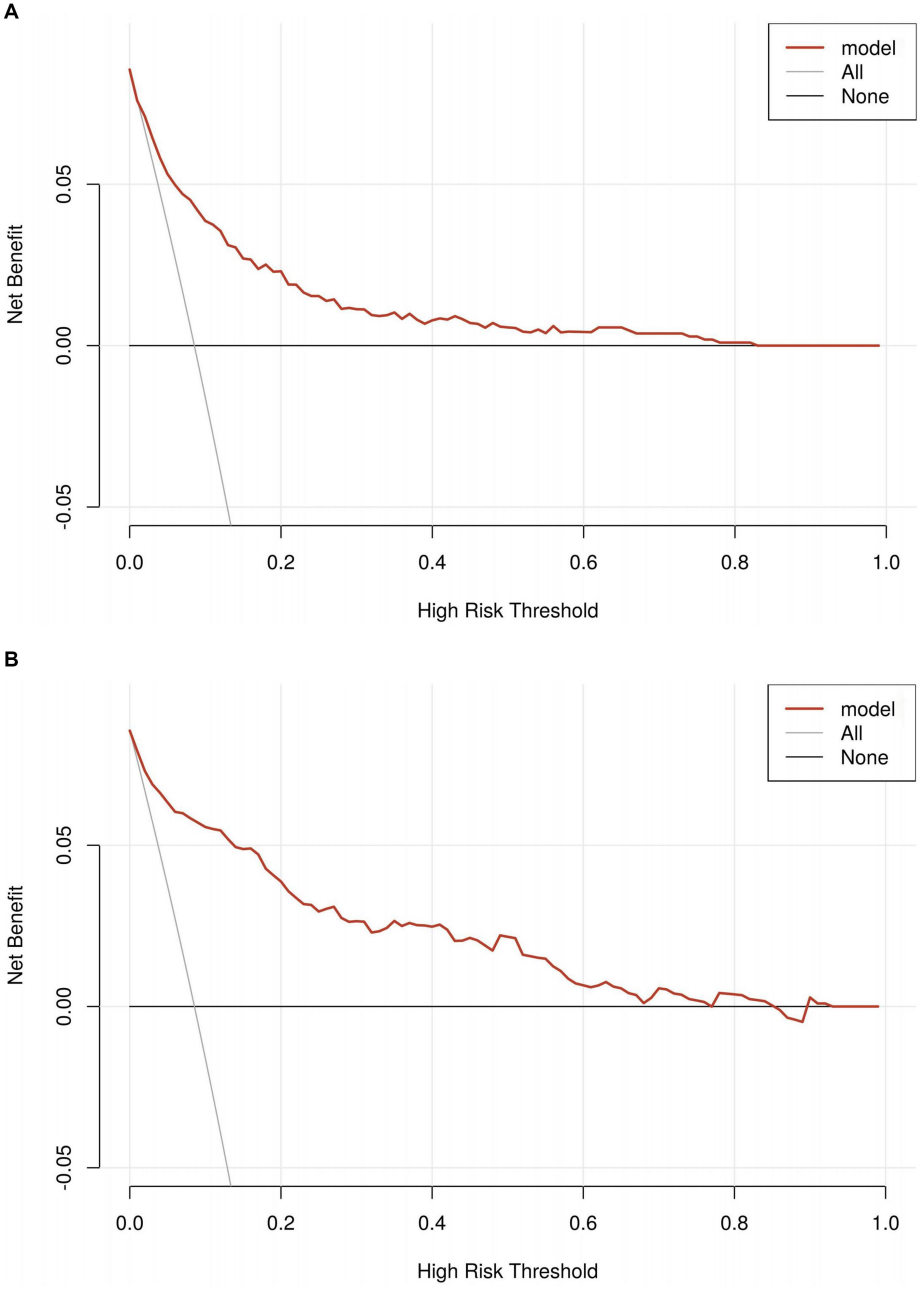
**(A)** The decision curve analyses in the training set. **(B)** The decision curve analyses in the validation set.

The predictive performance of the nomogram was evaluated using the following metrics: discrimination, the discriminative ability of the nomogram was assessed according to the AUC and the concordance index (C-index). In the training cohort, the nomogram achieved an AUC of 0.91 (95% CI 0.88–0.93) (Figure 3A), indicating excellent discriminative ability. The validation cohort demonstrated similar performance, with an AUC of 0.89 (95% CI 0.84–0.93) (Figure 3B). Calibration was evaluated by plotting calibration curves that compared the predicted probability of ICI with the observed outcomes. Both the training (Figure 4A) and validation (Figure 4B) cohorts demonstrated good agreement between the predicted and actual probabilities, as reflected by the non-significant Hosmer-Lemeshow goodness-of-fit test results (*p* > 0.05). The clinical utility of the nomogram was analyzed using DCA (Figures 5A,B), which calculated the net benefit of using the model across a range of threshold probabilities.

## Discussion

ICI (s) significantly increase the risk for persistent complications in patients undergoing cerebral aneurysm surgery (16). These infections impose substantial burdens on patients and their families. Identifying and understanding the relevant risk factors are essential for effective prevention and management. To the best of our knowledge, this is the first nomogram model developed to predict ICI after intracranial aneurysm surgery, demonstrating excellent predictive performance. To validate the accuracy and reliability of the model, multiple indicators were used to comprehensively evaluate its predictive capability for ICI after intracranial aneurysm surgery. In our study, 6 independent risk factors for postsurgical ICI were identified using multivariate logistic regression analysis. These included EVD, pneumonia, tracheotomy, and PCT, CRP, and Alb levels. Based on these factors, a nomogram prediction model was developed. This model serves as a practical and accessible tool for clinicians, aiding in risk stratification and guiding targeted interventions to mitigate the likelihood of ICI (s).

Consistent with previous studies, our results indicated that the EVD was a significant risk factor for postoperative ICI in patients undergoing intracranial aneurysm surgery. EVD disrupts the natural barriers of the central nervous system, creating a direct pathway for pathogens to enter the CSF (17). Furthermore, the presence of an indwelling catheter in the sterile environment of the brain provides a surface for biofilm formation, which enhances bacterial adherence and resistance to antimicrobial treatment (s) (18). To mitigate the risk for ICI, strict adherence to aseptic techniques during EVD placement and maintenance is essential (19). Routine monitoring for early signs of infection, timely removal of the EVD once it is no longer necessary, and considering prophylactic antibiotic regimens should also be prioritized. By implementing these measures, the likelihood of postoperative ICI can be significantly reduced, thereby improving the outcomes of patients with intracranial aneurysms (20).

Pneumonia was a significant risk factor for postoperative ICI in patients undergoing intracranial aneurysm surgery, underscoring its crucial role as an independent predictor in the postoperative period. Pneumonia is often associated with prolonged intubation, mechanical ventilation, and/or impaired cough reflex due to neurological deficits, and can lead to systemic inflammation and hematogenous dissemination of pathogens (21). These mechanisms contribute to the seeding of infectious agents in the CSF or surgical site, increasing the likelihood of ICI (22). To address this, vigilant respiratory management in the perioperative and postoperative phases is critical. Strategies such as early weaning from mechanical ventilation, effective pulmonary hygiene, and prevention of aspiration are paramount (23). Additionally, timely identification and treatment of pneumonia through clinical and radiological assessment, along with the judicious use of antibiotics, can help mitigate the cascade of infections leading to ICI. Recognizing and managing pneumonia as a modifiable risk factor is vital for improving surgical outcomes in this patient population (24).

Tracheotomy is one of the most effective interventions for maintaining airway patency in neurocritical patients with coma, airway obstruction, and/or respiratory impairment (25). However, this procedure disrupts the physiological barrier of the airway mucosa, directly exposing the previously sterile lower airway to external pathogens. This creates a direct pathway for bacteria to enter the airways, thereby increasing the risk for infection. In patients requiring mechanical ventilation, procedures such as tracheal intubation or tracheotomy inherently compromise the airway’s protective barriers (26). Prolonged pressure exerted by the tubing and the endotracheal cuff can physically damage the airway mucosa, leading to edema, congestion, erosion, and even necrosis (27). The risk for infection increases with the duration of mechanical ventilation. Tracheotomy further increases the risk for respiratory infections due to increased air exposure, which may also contribute to severe complications, including ICI. These risks underscore the need for meticulous care and monitoring of tracheotomized patients to minimize infection-related complications.

Aside from clinical characteristics, laboratory investigations in patients with intracranial aneurysms also indicate that ICIs may be more severe. This study found that CRP, PCT, and Alb levels were associated with the occurrence of ICI. Elevated CRP levels often reflect an acute-phase response to tissue injury, systemic inflammation (28), or infection, and persistently high postoperative CRP levels can indicate an increased risk for the development of ICI. CRP promotes the recruitment of immune cells to sites of injury or infection, contributing to the local inflammatory response (29). However, excessive or prolonged inflammation can compromise the blood–brain barrier, facilitating the entry of pathogens into the central nervous system. Additionally, elevated CRP levels are often associated with systemic conditions, such as pneumonia or urinary tract infections, which can further predispose patients to secondary intracranial infections. Elevated postoperative PCT levels are strongly associated with an increased likelihood of ICI because they reflect the presence of bacterial pathogens and the activation of systemic immune defenses (30). Unlike other inflammatory markers, such as CRP, PCT levels rise more rapidly in response to bacterial infections and decline quickly after effective treatment, providing a dynamic measure of infection status (31). Persistently elevated or rising PCT levels in the postoperative period may indicate ongoing infection or inadequate response to treatment, necessitating further diagnostic and therapeutic interventions. Alb level has been identified as a significant risk factor for postoperative ICI in patients undergoing surgery for intracranial aneurysms, underscoring its pivotal role in immune competence and physiological stability. Hypoalbuminemia, commonly observed in surgical and critically ill patients, is strongly associated with increased susceptibility to infections, including those caused by ICI. Low serum Alb levels may reflect poor nutritional status, systemic inflammation, or severe physiological stress, all of which compromise the body’s ability to mount an effective immune response (32). Hypoalbuminemia has also been linked to impaired wound healing and disruption of the blood–brain barrier, increasing the likelihood of pathogen invasion into the central nervous system. In the postoperative period, hypoalbuminemia may arise from surgical stress, fluid shifts, or inadequate nutritional intake (33). Patients with low Alb levels often experience prolonged recovery times, higher rates of complications, and an increased risk for secondary infections. Therefore, assessing and optimizing Alb levels is crucial for reducing the risk associated with ICI.

To the best of our knowledge, this study is the first to comprehensively explore risk factors and develop a predictive model for ICIs following intracranial aneurysm surgery. Notably, we constructed a robust nomogram that accurately estimated the likelihood of postoperative ICIs, offering an essential tool for early risk assessment and targeted clinical management. The nomogram demonstrated robust predictive accuracy, good calibration, and significant clinical utility, making it a valuable tool for personalized risk assessment of ICI following intracranial aneurysm surgery. It supports individualized risk evaluation and enhances clinical decision-making, ultimately improving patient outcomes.

### Limitations

This study has several limitations. First, the nomogram prediction model lacked external validation. To enhance reliability and generalizability, multicenter studies should be conducted to gather external datasets for validation, which would significantly bolster the credibility of the model. Second, findings from this investigation may have limited applicability to broader populations. Increasing the sample size and incorporating data from multiple centers would improve the generalizability of the model. Additionally, prolonged operative duration can contribute to higher infection risk due to extended tissue exposure, increased blood loss, and prolonged mechanical ventilation. We acknowledge that larger or more complex aneurysms may necessitate prolonged surgical duration, extensive tissue manipulation, and increased use of surgical adjuncts, all of which could indirectly elevate ICI risk. While we did not include these variables in our final predictive model, future studies should explore their role in infection risk stratification to enhance the accuracy and clinical applicability of ICI prediction. Furthermore, a key limitation is that all variables in the current nomogram were obtained postoperatively, limiting its value for preoperative risk assessment. Future studies should incorporate preoperative factors such as HH grade, aneurysm size and location, surgical duration, intraoperative rupture, and antibiotic use to improve early prediction and clinical utility. As such, to refine and validate the model further, a multicenter prospective study with a larger sample size is essential. Such efforts would help address potential confounding biases, enable identification of additional relevant risk factors and ultimately produce a more accurate and reliable predictive tool.

## Conclusion

The prediction nomogram model, based on 6 common clinical and laboratory test variables (pneumonia, EVD, tracheotomy, PCT, CRP, and Alb), was able to easily and accurately predict ICI after intracranial aneurysm surgery. The nomogram developed was a robust and practical tool for predicting the risk for ICI after intracranial aneurysm surgery. It demonstrated strong predictive accuracy and calibration with potential applications in identifying high-risk patients and guiding individualized preventive strategies. However, validation in a broader population is recommended to enhance the generalizability of the model.

## Data Availability

The raw data supporting the conclusions of this article will be made available by the authors, without undue reservation.

## Footnotes

^1^ http://www.R-project.org/
